# Recent advancements in the diverse roles of polymerase-associated proteins in the replication and pathogenesis of Newcastle disease virus

**DOI:** 10.1186/s13567-024-01429-0

**Published:** 2025-01-12

**Authors:** Jinghang Zhou, Yuqi Duan, Menglan Liu, Jinyang Liu, Zenglei Hu, Zhiqiang Duan

**Affiliations:** 1 Key Laboratory of Animal Genetics, Breeding and Reproduction in The Plateau Mountainous Region, Ministry of Education, Guizhou University, Guiyang, 550025 China; 2 Key Laboratory of Animal Genetics, Breeding and Reproduction of Guizhou Province, Guizhou University, Guiyang, 550025 China; 3 College of Animal Science, Guizhou University, Guiyang, 550025 China; 4 Joint International Research Laboratory of Agriculture and Agri-Product Safety, Ministry of Education, Yangzhou University, Yangzhou, 225009 China

**Keywords:** Newcastle disease virus, polymerase-associated protein, virulence, viral replication, pathogenesis

## Abstract

Newcastle disease virus (NDV) is a significant member of the *Paramyxoviridae* family, known for causing epidemics and substantial economic losses in the poultry industry worldwide. The NDV RNA genome primarily encodes six structural proteins (N, P, M, F, HN, and L) and two non-structural proteins (V and W). Among these, the polymerase-associated proteins (N, P, and L) and the viral RNA (vRNA) genome form the ribonucleoprotein complex, which plays a crucial role in the synthesis and transcription of NDV vRNA. In the last two decades, numerous studies have demonstrated that the polymerase-associated proteins are linked to the virulence, pathotype, and thermostability of NDV. Additionally, the interactions between these polymerase-associated proteins and host proteins are closely related to the NDV’s replication and pathogenicity. Despite significant progress in understanding the unique and shared functions of NDV polymerase-associated proteins, research on these viral proteins’ structure and function is less comprehensive than other NDV proteins, and the available information is often scattered. Therefore, this article systematically summarises and reviews the research progress made in understanding the structural features, virulence, pathotype, and thermostability correlation of NDV polymerase-associated proteins, as well as the critical roles of interactions between polymerase-associated proteins and host proteins in NDV replication and pathogenicity. This review aims to enhance our understanding of the complex functions of polymerase-associated proteins in NDV replication and pathogenesis and to contribute to the development of more effective vaccines and antiviral drugs against NDV challenges.

## Introduction

Newcastle disease (ND) is caused by infection with the virulent Newcastle disease virus (NDV), one of the most highly contagious diseases affecting avian species. It often leads to epidemics and significant economic losses worldwide [1]. NDV, also known as avian paramyxovirus 1 (APMV-1) or avian orthoavulavirus 1 (AOAvV-1), belongs to the genus *Orthoavulavirus* within the family *Paramyxoviridae.* Currently, it is categorised into two classes, Class I and Class II, based on the recent phylogenetic classification system, although there is only one serotype among existing NDV strains (NDVs) [2, 3].

NDVs are primarily divided into three pathotypes-velogenic, mesogenic, and lentogenic-based on their pathogenicity in chickens [4]. Class I NDVs are mostly isolated from wild birds and posess a genome length of 15 198 nt, with a lentogenic pathotype [5]. In contrast, Class II NDVs are generally isolated from various bird species and have genome sizes ranging from 15 186 to 15 192 nt, exhibiting velogenic, mesogenic, and lentogenic pathotypes [6, 7].

Notably, some Class I NDVs show varying levels of increased virulence and replication ability due to conventional start codon mutations (ACG, CTA, and ATA) in the *F* gene. The ATA mutation, located at genomic positions 4523 to 4525 nt (the authentic start codon in the *F* gene of Class I NDVs) is a key determinant [8]. Interestingly, this mutation at the corresponding site of the Class II genotype II LaSota does not affect virulence and replication ability in chickens. This suggests that specific mutations in the *F* gene may be a specific characteristic of Class I NDVs, and the ongoing persistent transmission and circulation of Class I NDVs might lead to the emergence of more virulent strains [8].

Additionally, numerous studies have reported that the natural hosts for NDVs have expanded from avian species to non-avian species, including calves [9], hamsters [10], swine [11], and even humans [12–14]. This shift suggests that NDV may pose a zoonosis risk that needs attention [15, 16]. Also, NDV has been used extensively not only as a nonpathogenic oncolytic virus and a promising vector vaccine against multifarious pathogens [17, 18] but also as a model virus to understand better the replication and pathogenicity of related paramyxoviruses [19, 20]. Consequently, advancements in the fundamental biology of NDV have garnered significant research interest.

**Figure 1:**
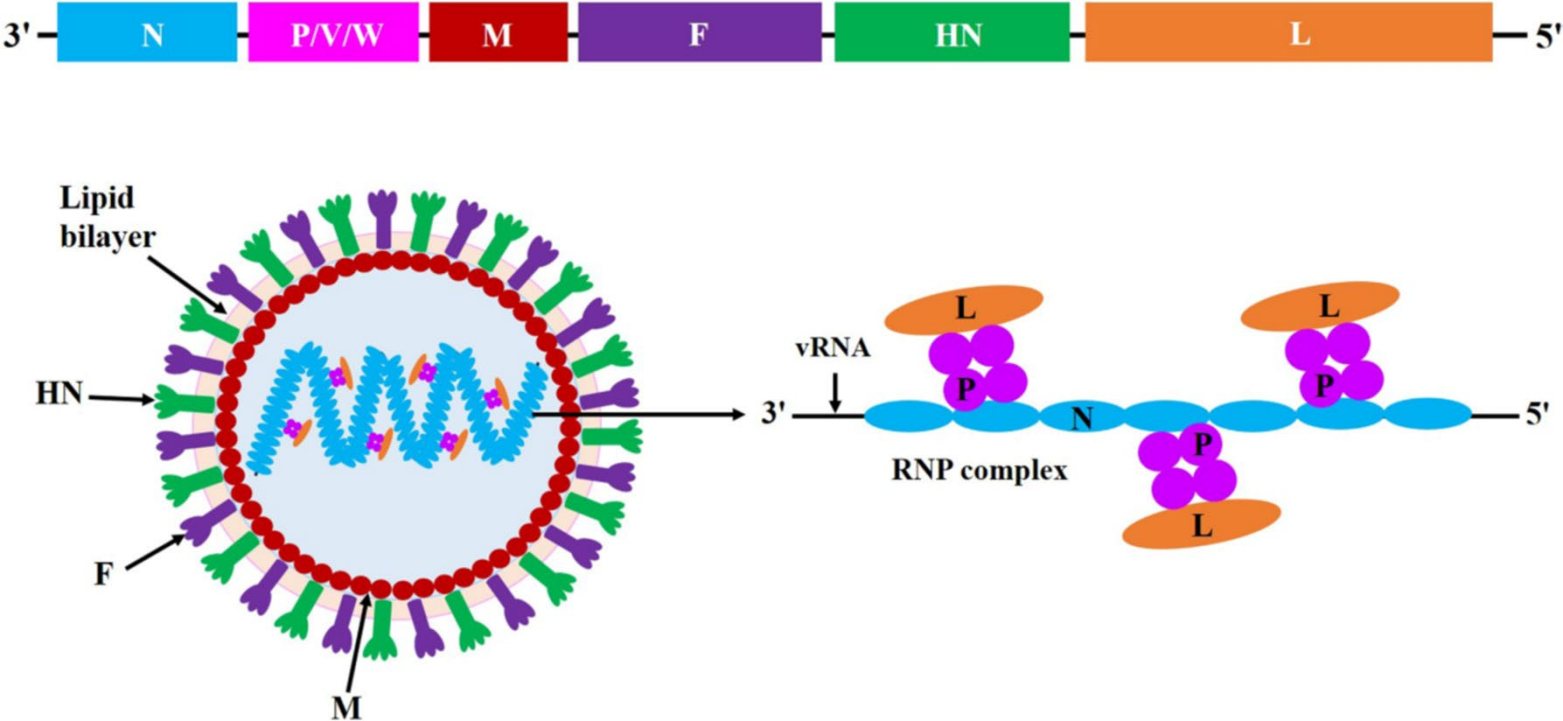
**Schematic representation of NDV genome and virion structure.** The RNA genome of NDV contains six major genes that encode six structural proteins (N, P, M, F, HN, and L) in the order 3′–N–P/V/W–M–F–HN–L–5′, and two non-structural proteins (V and W) due to the RNA editing of *P* gene mRNA. Among these viral proteins, the F, HN, and M proteins tightly surround the outer and inner surface of the viral envelope, respectively, and the N, P, and L proteins, together with vRNA, are found inside the virions to form an RNP complex. The lipid bilayer envelope of NDV virion is shown as light orange.

NDV is an enveloped virus characterised by a non-segmented, single-stranded, negative-sense RNA genome. This genome contains six significant genes that encode six structural proteins, N, P, M, F, HN, and L, in the following order: 3′-N-P/V/W-M-F-HN-L-5′. Additionally, two non-structural proteins, V and W, are produced as a result of mRNA editing of the viral *P* gene (Figure 1).

Among the NDV proteins, the envelope-associated proteins (M, F, and HN) tightly surround the inner and outer surfaces of the viral envelope, respectively, while the polymerase-associated proteins (N, P, and L) are found inside the virions. These polymerase proteins form the ribonucleoprotein (RNP) complex along with the viral RNA (vRNA) genome (Figure 1) [21]. Notably, the non-structural proteins V and W are present in very small quantities in NDV virions and do not contribute to the assembly of virions [22].

Much has been understood about the biological functions of NDV proteins in the viral life cycle. Specifically, the N protein encapsidates the vRNA into helical N-vRNA complexes known as nucleocapsids (NC). This encapsidation protects vRNA from degradation by nucleases and prevents host immune responses from recognising the vRNA. Additionally, the N protein assists in vRNA genome replication, transcription, and virion maturation [23, 24]. The P protein acts as a bridge connecting the N and L proteins and facilitates the release of the N protein from RNP, exposing vRNA. This exposure allows the L protein to initiate the replication and transcription of vRNA [25]. The L protein plays a key role in the RNA-dependent RNA polymerase (RdRp) activity, essential for the 5′ capping, methylation, and polyadenylation of newly synthesised viral mRNAs [26]. In summary, the NDV polymerase-associated proteins are vital for the replication and transcription of the vRNA genome.

Evidence suggests that virulence is positively correlated with the replication efficiency and pathogenicity of NDV [27–29]. To date, numerous studies have been conducted to investigate the virulence of NDV using reverse genetics techniques. The consensus is that the envelope glycoproteins F and HN serve as the primary virulence determinants by influencing tissue tropism, cell fusion, and the pathogenic phenotypes of NDV [27, 30, 31]. Additionally, the M, V, and W proteins contribute to NDV virulence by playing crucial roles in promoting viral morphogenesis and antagonising the interferon response, respectively [32–35].

Regarding the polymerase-associated proteins, the homologous N, P, and L proteins significantly impact NDV virulence by affecting RNA polymerase activity and vRNA transcription and replication [27]. Recent evidence indicates that these polymerase-associated proteins have a strong correlation with the virulence and pathotype of NDV through specific distinctive amino acid (aa) sites, motifs, and regions. Furthermore, studies have also shown that polymerase-associated proteins are linked to NDV’s thermostability and are essential for viral immune evasion, RNA polymerase activity, and viral protein synthesis through their interactions with host proteins.

These findings highlight the multifaceted roles of polymerase-associated proteins in the replication and pathogenicity of NDV. Over the last two decades, although much research has focused on clarifying the structure and function of NDV polymerase-associated proteins, the available reference information remains scattered. Therefore, to gain a deeper understanding of the roles of these proteins in NDV replication and pathogenesis, a systematic summary and review of their structural features, virulence, pathotype, and thermostability correlations, as well as the essential roles of polymerase-associated protein-host protein interactions, have been conducted. The accumulative knowledge from this research not only enhances our understanding of the molecular mechanisms underlying the complex replication and pathogenicity of NDV but also provides valuable insights for the effective prevention and control of NDV and related paramyxoviruses.

## Structural features of NDV polymerase-associated proteins

### Structural features of the NDV N protein

The open reading frame (ORF) of the *N* gene derived from various NDVs is 1,470 nt long and encodes a protein of 489 aa with a molecular weight of approximately 55 kilodaltons (kDa). The N protein is the most abundant viral protein found in NDV-infected cells and NDV virions. This protein can selectively bind to genomic and antigenomic vRNA to form helical NC structures [24].

Interestingly, the encapsidation of RNA by the N protein does not depend on specific nt sequences. The NDV N protein, expressed in bacteria in the absence of virus infection, can also form NC-like structures due to its non-specific binding of cellular RNAs [24]. Research has shown that the NDV N protein primarily exists in a soluble, monomeric form known as N^0^. In this form, the newly synthesised N protein is bound to the P protein, creating an N^0^-P complex that prevents inappropriate self-assembly of the N^0^ protein [36, 37].

**Figure 2:**
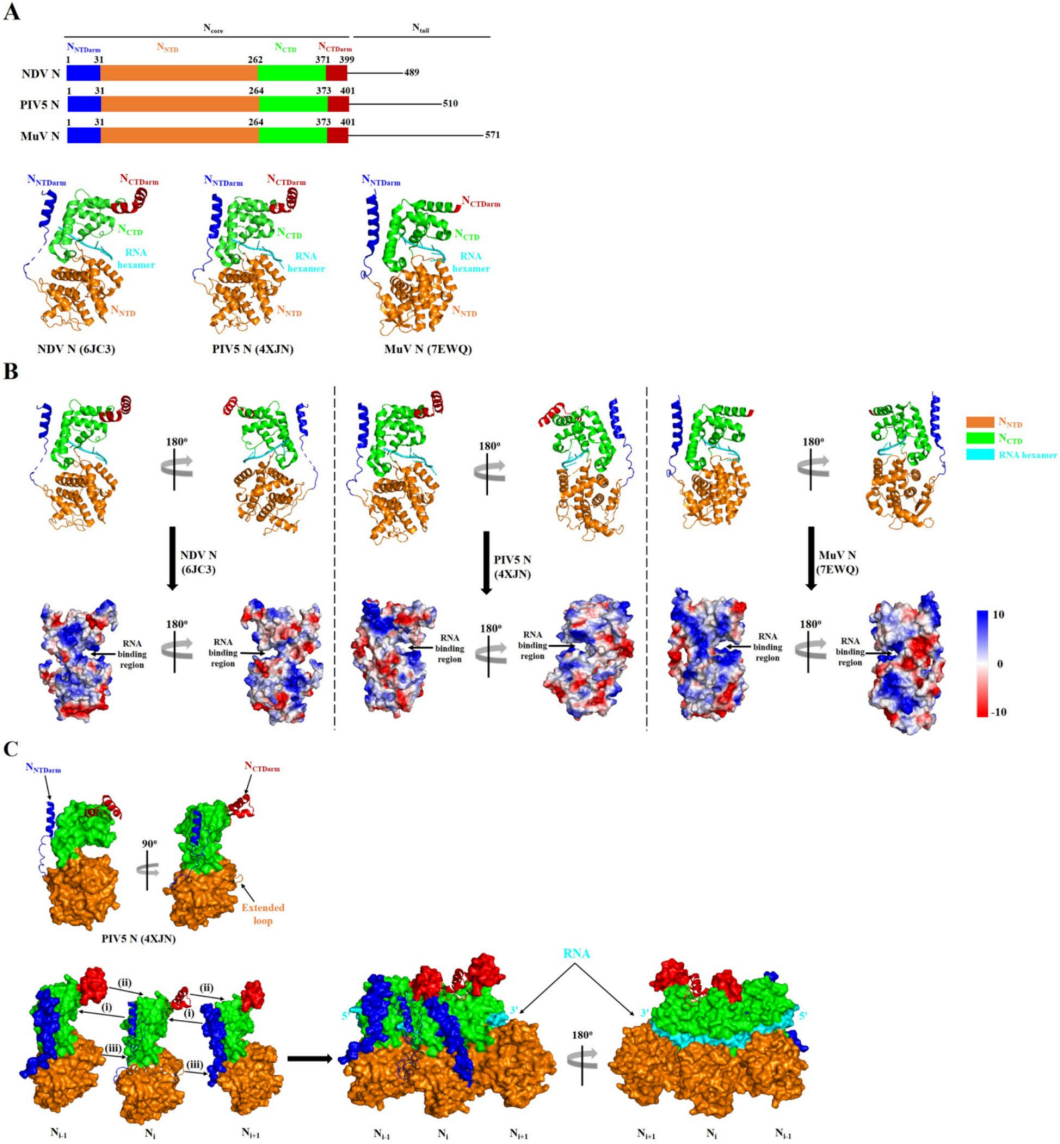
**The structure of NDV, PIV5, and MuV N proteins.** **A** Organisation and structure of the NDV, PIV5, and MuV N proteins. The structures of N_NTDarm_, N_NTD_, N_CTD_, and N_CTDarm_ in the cartoon drawing are shown in blue, orange, green, and red, respectively. The RNA hexamer binding to the N protein is shown in cyan. **B** Schematic representation of the positively charged groove (blue) between the N_NTD_ and N_CTD_ domain of NDV, PIV5, and MuV N proteins binding to a negatively charged RNA hexamer. **C** Surface representation of three PIV5 N protomers with the N_NTDarm_ (blue), N_CTDarm_ (red), and extended loop (orange) of the N_i_ protomer shown in the cartoon representation. The RNA is shown in cyan. The above structure representations are generated using the software PyMoL 2.5.5.

It is well-known that the N proteins of NDV and other paramyxoviruses, such as the parainfluenza virus 5 (PIV5) and the mumps virus (MuV), have two major regions. These regions include a highly conserved n-terminal region (N_core_) and a disordered c-terminal region (N_tail_). The N_core_ consists of two domains: the n-terminal domain (N_NTD)_ and the c-terminal domain (N_CTD_), which are flanked arms known N_NTDarm_ and N_CTDarm_ (Figure 2A).

Despite showing low aa homology, the N proteins of NDV, PIV5, and MuV exhibit similar structural features (Figure 2A). Functional analyses indicate that the N_core_ is responsible for the N protein’s self-assembly and binding to vRNA, allowing the formation of helical NC structures. The N_tail_, on the other hand, primarily serves to connect the P and L proteins, forming the N-vRNA-RdRp complex necessary for vRNA synthesis and transcription [38, 39].

Additionally, like the N proteins of PIV5 and MuV [40, 41], the NDV N protein displays flexibility between the N_NTD_ and N_CTD_ domains, facilitating the presentation of RNA hexamers and allowing access for the viral polymerase when required (Figure 2A) [37]. Notably, a positively charged groove forms between the N_NTD_ and N_CTD_ domains, efficiently accommodating the encapsidated vRNA genomes of NDV, PIV5, and MuV (Figure 2B) [37, 40, 41]. Furthermore, lateral contacts between the N_NTDarm_ and the N_CTDarm_ of successive viral promoters contribute to the formation of an oligomeric helical NC structure [37, 40, 41].

To date, the N protein structures of NDV, PIV5, and MuV have been shown to exist in a high-order assembly state instead of a monomeric state. For example, analysis of the three-dimensional structure of the PIV5 N protein reveals that each N protomer forms tight connections with its neighbours through its two arms that protude from each side of the N protein (Figure 2C) [40].

On the one hand, the N_NTDarm_ of the N_i_ promoter forms an α-helix that binds to a hydrophobic groove located on the back of the N_CTD_ of the N_i-1_ promoter (Figure 2Ci). On the other hand, the N_CTDarm_ of the N_i_ promoter folds into two α-helixes that interact with the top of the N_CTD_ of the N_i+1_ promoter (Figure 2Cii). Additionally, an extended loop in the N_NTD_ of the N_i_ protomer inserts into a hole formed by the N_NTD_ and N_NTDarm_ of the N_i+1_ promoter (Figure 2Ciii) [40]. These N–N interactions curve the N oligomer, exposing the RNA on the outside of the helix while concealing all the tight inter-protomer connections inside the helix (Figure 2C) [40].

This mode of assembly is also observed in the N proteins of NDV and MuV. However, unlike the ring-like NC structure found in PIV5 and MuV N proteins [40, 41], the NDV N protein forms a single-turn spiral structure due to the superaddition of an upward shift of ~4.6 Å [37]. Each single-turn spiral consists of a total of 13 NDV N molecules, and each single-turn spiral has the potential to grow into a doubled-headed filament along a helical trajectory [37].

Further analysis indicates that two single-turn spirals are arranged in a back-to-back pattern to form a clam-shaped NC structure of NDV N protein. This clam-shaped core can serve as a seed for the growth of filaments [37]. Importantly, in both the clam-shaped structure and the resulting double-headed filament, the self-capping interface arises from loops (aa 114–120) of vertically adjacent NDV N proteins in the clam-shaped core. However, this Loop_114-120_ region is only involved in the assembly of the clam-shaped core and not in the helical assembly of the double-headed filament [37].

Moreover, the formation of the clam-shaped structure mediated by Loop_114-120_ is crucial for the replication, transcription, and translation of the vRNA genome [37]. Notably, helical, ring-like, and clam-shaped NC structures are present in the N proteins of *Paramyxoviridae* family, which includes the measles virus (MeV), cetacean morbillivirus (CeMV), PIV5, MuV, NDV, Sendai virus (SeV), and Nipah virus (NiV) [42, 43]. However, clam-shaped NC structures have only been observed in NDV, SeV, and NiV [37, 44, 45].

The detection of this structure in paramyxoviruses from different genera and its presence in NC extracted from NDV and SeV virions suggests its biological relevance [37, 44]. Evidence indicates the clam-shaped structure can play roles in seeding the assembly of double-headed helices, protecting the 5' end of the vRNA genome from nuclease degradation, and promoting the encapsidation of multiple nucleocapsids per virion [37, 46–48]. Together, these findings enhance our understanding of paramyxovirus NC structures' structural and functional diversity and inspire new approaches to antiviral drug development by targeting the N protein.

### Structural features of the NDV P protein

The ORF of the *P* gene derived from different strains of NDVs exhibits two length variations: one consisting of 1188 nt and the other 1,200 nt, respectively. These variants encode the proteins of 395 and 399 aa, respectively, with molecular weights ranging from 50 to 55 kDa [49]. The variation in molecular weights primarily stems from the presence of multiple phosphorylated forms at specific serine (S) and threonine (T) residues within the viral P protein [50].

While the NDV P protein lacks intrinsic enzymatic activity, it serves as a crucial link between N-vRNA and the L protein, essential for the replication and transcription of the vRNA genome. Therefore, it is an integral component of the viral RNP complex [51].

**Figure 3:**
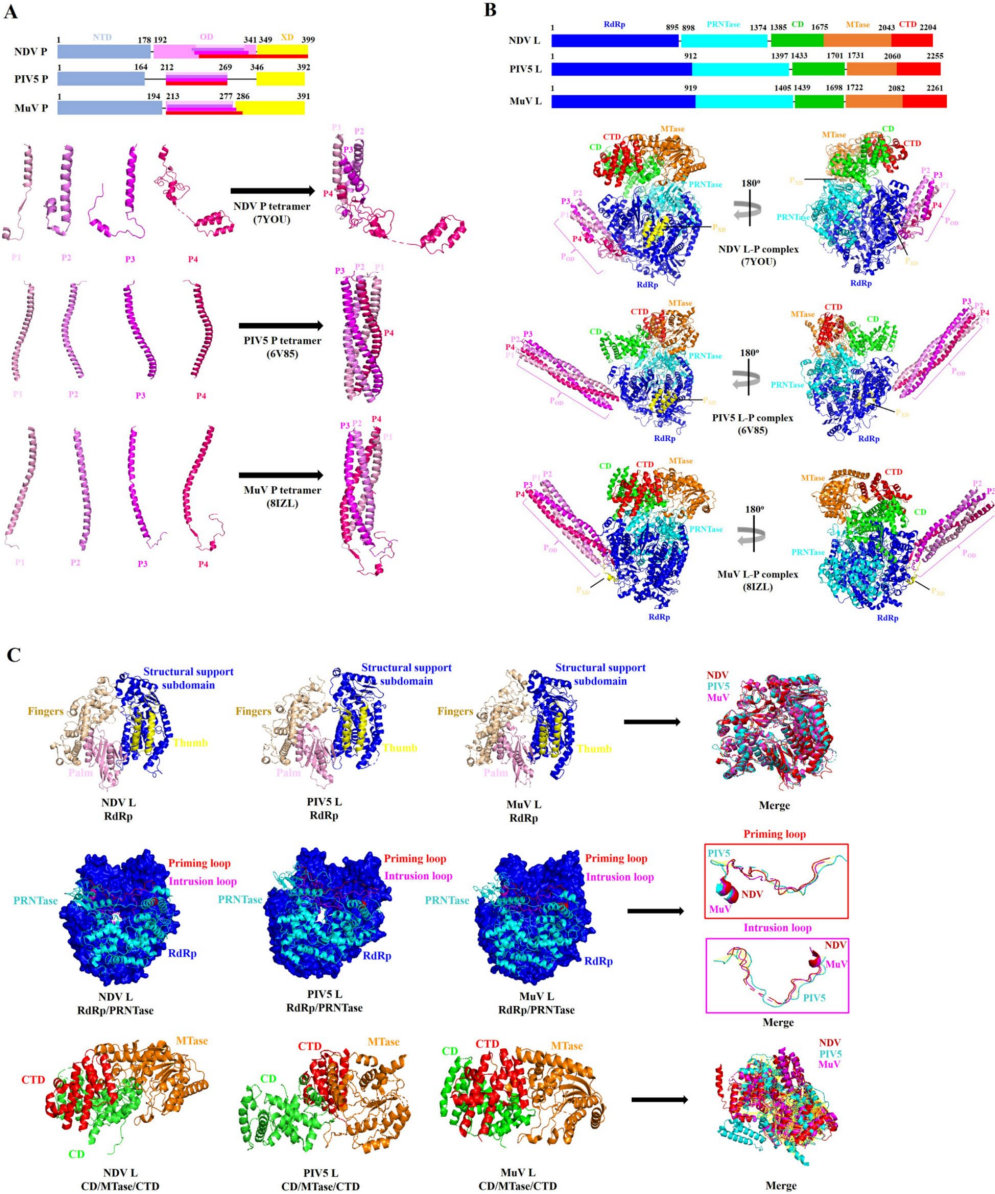
**The structure of NDV, PIV5, and MuV P and L proteins.** **A** Organisation and structure of the NDV, PIV5, and MuV P proteins. The P1 monomer (pink), P2 monomer (violet), P3 monomer (magenta), and P4 monomer (hot pink) constitute the tetrameric structure of the NDV, PIV5, and MuV P proteins, respectively. **B** Organisation of the NDV, PIV5, and MuV L proteins, and the cartoon drawing of the overall structure of NDV, PIV5, and MuV L-P complexes. The structures of the NDV, PIV5, and MuV L-P complexes in the cartoon drawing are coloured by domains, and the colouring scheme is identical to that in (**A** and **B**). **C** The structures of the RdRp, RdRp/PRNTase, and CD/MTase/CTD domains in the NDV, PIV5, and MuV L proteins. The superimposed structures of the RdRp, priming loop, intrusion loop, and CD/MTase/CTD among NDV, PIV5, and MuV L proteins are shown on the right. The above structure representations are generated using the software PyMoL 2.5.5.

Although the P proteins of paramyxoviruses, including NDV, PIV5, and MuV, do not share significant sequence homology, they do exhibit some common structural domains that perform specific functions. For instance, with NDV, the P proteins contain an inherently disordered N-terminal domain (NTD), a central oligomerisation domain (OD), and a C-terminal X domain (XD) (Figure 3A) [52–54].

Research has shown that the first 40–50 aa of the NDV P_NTD_ are responsible for binding to the nascent N^0^ protein, preventing it from randomly encapsidating non-specific vRNA [52, 55]. The NDV P_OD_ mainly facilitates the formation of a stable L–P complex by binding to the L protein, which is crucial for the interaction between vRNA and the L protein [52]. In contrast, the NDV P_XD_, plays a key role in helping the vRNA enter the template entrance channel and assists nucleoside triphosphates (NTPs) in entering the NTP entrance channel by interacting with the viral RNP [52].

Notably, the P_OD_ of NDV, PIV5, and MuV binds to and stabilises the L protein. In contrast, the P_XD_ and P_NTD_ regions of the P protein can sway irregularly to perform different functions during vRNA synthesis [52]. This behaviour is similar to findings reported for the P protein of human metapneumovirus (HMPV) P protein [56].

The cryo-electron microscopy (cryo-EM) structures of the P proteins from NDV, PIV5, and MuV have revealed distinct tetrameric conformations (Figure 3A) [52–54]. While these viral P proteins exhibit different four-helix bundle structures, they can interact extensively with the RdRp domain of the L protein (Figure 3B) [52–54]. Recently, a high-resolution tetrameric structure of the NDV P protein has been characterised, consisting of four monomers: P1 (aa 261–301), P2 (aa 250–305), P3 (aa 259–312), and P4 (aa 274–399) [52]. However, unlike the P4 monomer of PIV5 and MuV P tetramers, the P4 monomer of NDV P tetramer consists of the longest aa residues and contains the flexibility structure of P_XD_ (Figure 3A).

In the tetrameric structure of the NDV P protein, the regions (aa 261–289) of the P1 and P2 monomers, along with the regions (aa 349–399) of the P4 monomer, directly interact with the L protein. Meanwhile, the regions (aa 260–312) of the P3 monomer and the regions (aa 274–283 and 308–315) of the P4 monomer closely bind to the helices of P1 and P2 monomers (Figures 3A and B) [52]. This binding pattern differs from those observed in PIV5 and MuV P tetramers with their respective L proteins [53, 54].

Additionally, studies indicate that the L-binding P_XD_ located on the NTP entry side for NDV, PIV5, MuV, and HMPV belongs to the C-terminal domains of the P4, P2, P3, and P1 monomers, respectively [52–54, 56]. This suggests that the L-binding P_XD_ model among paramyxoviruses has diverse origins, and the structures of their P monomers may display different functional states during vRNA synthesis. Together, these findings indicate that the P protein of NDV and other paramyxoviruses has a unique tetrameric structure and interaction model with the L protein, which enhances our understanding of paramyxovirus vRNA synthesis.

### Structural features of the NDV L protein

The *L* gene has an ORF that is 6,615 nt long, encoding a protein of 2,204 aa with a molecular weight of approximately 250 kDa. Similar to the functional domains found in the L proteins of PIV5 and MuV [53, 54], the L protein of NDV contains five distinct domains: the RdRp domain (aa 1–895), the poly-ribonucleotide transferase domain (PRNTase) (aa 898–1374), the connector domain (CD) (aa 1385–1675), the methyltransferase domain (MTase) (aa 1676–2043), and the C-terminal domain (CTD) (aa 2044–2204) (Figure 3B) [52].

While the L is capable of RNA polymerase activity independently, the activity is significantly enhanced in the presence of the P protein [57]. Studies on the L protein from vesicular stomatitis virus (VSV), a representative RNA virus, indicate that binding between the NDV P protein and the L protein is essential for establishing a stable interaction between the CD/MTase/CTD module and the RdRp/PRNTase module [58, 59].

The formation of the L-P complex is crucial for efficient vRNA synthesis and transcription in paramyxoviruses [60–62]; however, there are notable differences in interaction patterns between the L and P proteins of NDV, PIV5, and MuV [52–54]. Recent research has shown that the interface area of interaction between the NDV L and P proteins is extensive and is mediated through hydrophobic and electrostatic interactions, as well as hydrogen bonds [52]. The four-helix bundles of NDV P_OD_ utilise the P1 monomer (aa 276–282) and the P2 monomer (aa 271–286) helixes to attach to the RdRp domain of the L protein. The flexible regions of the P1 monomer (aa 284–299) and the P2 monomer (aa 287–296) encircle the surface of the L protein, stabilising the P tetramer on the fingers subdomain of the L protein (Figure 3B and C).

Additionally, the residues K284/D287 of the P1 monomer and the residues D298/R300 of the P2 monomer form electrostatic interactions with the residues D385/R650/R714 and K416/H418/Y421 of the NDV L protein. The residues I285/L299 of the P2 monomer form hydrophobic interactions with E451/H659 of the NDV L protein [52].

Furthermore, a three-helix bundle derived from the region (aa 351–399) of the P4 monomer is located near the NTP entrance channel and interacts with the palm subdomain of NDV L protein. Within the P4 monomer, four residues—R356/D357/R366/K393—establish electrostatic interactions with E301/D309/H336 of the L protein. Only one residue, L362, forms hydrophobic interactions with L299/F306 of the L protein [52].

In contrast to the interaction interface between the L and P proteins of NDV and MuV, the interaction region between PIV5 L and P proteins does not appear to involve a large interface, likely due to the low resolution (Figure 3B). Therefore, a more precise structure of the PIV5 L-P complex may be necessary to understand better the potential mechanisms for spatial–temporal regulation of vRNA synthesis.

Research has demonstrated that the RdRp domain of the NDV L protein comprises four subdomains: fingers, palm, thumb, and a structural support subdomain. This arrangement exemplifies a typical and highly conserved “fingers-palm-thumb” right-hand fold, similar to that of the PIV5 and MuV L proteins (Figure 3C). Six specific residues (R552, I553, Y645, G750, D751, and N752) have been identified as critical for the RdRp activity of the NDV L protein [52].

Similar to the PRNTase domains of PIV5 and MuV L proteins [53, 54], the PRNTase domain of the NDV L protein contains structures known as the priming loop and the intrusion loop (Figure 3C). The priming loop (aa 1186–1216) is directly opposite to the RdRp active site and typically adopts an RNA elongation conformation. In contrast, the intrusion loop (aa 1257–1289) is located near the RdRp active sites [52].

Unlike the conserved RdRp/PRNTase domains, the CTD domain connects with the CD and MTase domains to form the CD/MTase/CTD module. This module exhibits a unique organisation and a highly flexible structure among the NDV, PIV5, and MuV L proteins (Figure 3B and C) despite the relative similarity in their individual structures [52–54]. Notably, the MTase domain of NDV, PIV5, and MuV L proteins contains catalytic motifs (G-D-N and K-D-K-E) that are essential for vRNA synthesis through 5' capping and methylation of viral mRNAs. This indicates a high level of conservation of MTase domain in the *Paramyxoviridae* family [55, 57, 61].

Furthermore, the paramyxovirus NDV and PIV5 L proteins have template entrance, NTP entrance, template exit, and product exit channels. These are crucial for vRNA synthesis and transcription [63]. However, although these channels are open in both the NDV and PIV5 L proteins, the MTase/CTD module of NDV exhibits a 70° rotation relative to the RdRp/PRNTase module, positioning it opposite to the CD domain of PIV5 [52, 53]. Unlike the CD/MTase/CTD module in PIV5 and MuV L proteins [53, 54], the CD domain of NDV L protein is only loosely associated with its RdRp/PRNTase module. Consequently, movement of the NDV CD domain can trigger structural rearrangements of the MTase/CTD module in the L-P complex [52].

These findings suggest that the CD/MTase/CTD modules of paramyxovirus L proteins adopt different arrangements, and that the rearrangement of this module may be crucial for transitioning from the initiation state to the elongation state during paramyxovirus vRNA synthesis.

## Correlation between polymerase-associated proteins and NDV virulence

NDVs can be classified into three pathotypes: lentogenic, mesogenic, and velogenic, based on the severity of infection in chickens [4]. The specific virulence determinants that explain the differences in pathogenicity among these NDV pathotypes are not fully understood. Most studies agree that the surface glycoproteins F and HN are the primary virulence factors for NDV [27, 28, 31, 64]. In addition, the roles of polymerase-associated proteins in NDV virulence have been widely investigated using reverse genetics techniques (Table 1).

**Table 1:** **Properties of recombinant NDVs harboring polymerase-associated protein gene exchange**

Virus	Parent	Genotype	Pathotype^*^	MDT^†^	ICPI	Reference
rBC	Beaudette C	II	Mesogenic	62 h	1.45	[65]
rBC(N)^LaSota^			Mesogenic	60 h	1.31	
rBC(P)^LaSota^			Mesogenic	64 h	1.24	
rBC(L)^LaSota^			Velogenic	53 h	1.70	
rBC(N + P)^LaSota^			Mesogenic	58 h	1.44	
rLaSota	LaSota	II	Lentogenic	106 h	0.00	
rLaSota(N)^BC^			Lentogenic	109 h	0.00	
rLaSota(P)^BC^			Lentogenic	114 h	0.00	
rLaSota(L)^BC^			Lentogenic	115 h	0.00	
rLaSota(N + P)^BC^			Lentogenic	108 h	0.00	
rAV324	AV324/96	VI	Lentogenic	nd	0.10	[66]
rAV324(N)^Herts^			Lentogenic	nd	0.04	
rAV324(P)^Herts^			Lentogenic	nd	0.25	
rAV324(L)^Herts^			Lentogenic	nd	0.48	
rAV324(N + P)^Herts^			Lentogenic	nd	0.70	
rAV324(N + P + L)^Herts^			Mesogenic	nd	1.03	
rHerts	Herts/33	IV	Velogenic	nd	1.54	
rHerts(N)^AV324^			Mesogenic	nd	1.35	
rHerts(P)^AV324^			Mesogenic	nd	1.33	
rHerts(L)^AV324^			Mesogenic	nd	1.30	
rHerts(N + P)^AV324^			Mesogenic	nd	1.35	
rHerts(N + P + L)^AV324^			Lentogenic	nd	0.55	
rBC	Beaudette C	II	Velogenic	nd	1.58	[67]
rBC(N)^GBT^			Velogenic	nd	1.62	
rBC(P)^GBT^			Velogenic	nd	1.60	
rBC(L)^GBT^			Velogenic	nd	1.71	
rBC(N + P + L)^GBT^			Velogenic	nd	1.75	
rGBT	GB Texas	II	Velogenic	nd	1.91	
rGBT(N)^BC^			Velogenic	nd	1.88	
rGBT(P)^BC^			Velogenic	nd	1.90	
rGBT(L)^BC^			Velogenic	nd	1.83	
rGBT(N + P + L)^BC^			Velogenic	nd	1.78	
rSG10	SG10	VII	Velogenic	57 h	1.86	[68]
rSG10(N)^LaSota^			Velogenic	72 h	1.78	
rSG10(P)^LaSota^			Velogenic	52 h	1.89	
rSG10(L)^LaSota^			Velogenic	83 h	1.68	
rSG10(N + P)^LaSota^			Velogenic	58 h	1.90	
rSG10(N + L)^LaSota^			Mesogenic	86 h	1.48	
rSG10(P + L)^LaSota^			Mesogenic	84 h	1.40	
rSG10(N + P + L)^LaSota^			Mesogenic	89 h	1.32	
rLaSota	LaSota	II	Lentogenic	> 120 h	0.00	
rLaSota(N)^SG10^			Lentogenic	> 120 h	0.00	
rLaSota(P)^SG10^			Lentogenic	> 120 h	0.00	
rLaSota(L)^SG10^			Lentogenic	> 120 h	0.00	
rLaSota(N + P)^SG10^			Lentogenic	> 120 h	0.34	
rLaSota(N + L)^SG10^			Lentogenic	> 120 h	0.14	
rLaSota(P + L)^SG10^			Lentogenic	> 120 h	0.12	
rLaSota(N + P + L)^SG10^			Lentogenic	> 120 h	0.63	
rI4	I4	VII	Velogenic	nd	1.96	[69]
rI4(N)^Herts^			Velogenic	nd	1.90	
rI4(P)^Herts^			Velogenic	nd	1.85	
rI4(L)^Herts^			Velogenic	nd	1.80	
rI4(N + P + L)^Herts^			Velogenic	nd	1.72	
rHerts	Herts/33	IV	Velogenic	nd	1.65	
rHerts(NL)^I4^			Velogenic	nd	1.69	
rHerts(P)^I4^			Velogenic	nd	1.70	
rHerts(L)^I4^			Velogenic	nd	1.77	
rHerts(N + P + L)^I4^			Velogenic	nd	1.88	
rJS5/05	JS5/05	VII	Velogenic	46 h	1.88	[70]
rJS5/05(N + P + L)^Herts^			Velogenic	43 h	1.82	
rHerts	Herts/33	IV	Velogenic	48 h	2.00	
rHerts(N + P + L)^JS5/05^			Velogenic	49 h	1.80	

^*^Recombinant NDVs with an ICPI value > 1.60 are defined as velogenic, an ICPI value of 0.60 to 1.50 is mesogenic, and an ICPI value < 0.50 is lentogenic.

^†^nd, indicates that MDT is not detected.

One study involved exchanging individual *N*, *P*, and *L* genes between a mesogenic NDV (Beaudette C, genotype II) and a lentogenic NDV (LaSota, genotype II). The findings revealed that the mean death time (MDT) and intracerebral pathogenicity index (ICPI) values of the N and/or P chimeric recombinant NDVs aligned with those of their respective parental viruses, suggesting that the N and P proteins play a minor role in NDV virulence (Table 1) [65]. In contrast, substituting the *L* gene from rBC with the *L* gene from rLaSota significantly increased the ICPI value of rBC(L)^LaSota^, indicating that the L protein has a significant impact on NDV virulence (Table 1) [65].

Another similar investigation, involved exchanging viral genes between a velogenic NDV (Herts/33, genotype IV) and a lentogenic NDV (AV324/96, genotype VI). This study confirmed that while the L protein is related to NDV virulence, the combined influence of the N, P, and L proteins has the greatest impact on NDV virulence (Table 1) [66]. Additional research involving viral gene exchanges between velogenic NDV strains (Beaudette C, genotype II/GB Texas, genotype II; I4, genotype VII/ Herts/33, genotype IV) or between a velogenic NDV strain (GS10, genotype VII) and a lentogenic NDV strain (LaSota, genotype II) also supported the conclusion that the L protein is closely linked to NDV virulence, which can be further enhanced by homotypic N, P, and L proteins (Table 1) [67–69].

Interestingly, when polymerase-associated protein genes were exchanged between two velogenic NDVs [rJS5/05(N + P + L)^Herts^ and rHerts (N + P + L)^JS5/05^], there was no significant impact on virulence, replication, or pathogenicity of recombinant NDVs compared to their respective parent viruses [70]. This finding differs from gene exchange results from other velogenic NDVs [67, 69]. A possible explanation for this discrepancy is that the RNA polymerase activity of the N, P, and L proteins is directly associated with NDV virulence [65, 66, 68]. In the case of the polymerase-associated proteins of JS5/05 and Herts/33, both exhibited equivalent RNA polymerase activities [70].

Overall, these findings indicate that the homologous N, P, and L proteins are closely linked to NDV virulence, primarily by influencing RNA polymerase activity and vRNA transcription and replication.

## Correlation between polymerase-associated proteins and NDV pathotype

Current evidence demonstrates that the reverse genetics technique allows for extensive modification of the NDV genome, providing more significant opportunities to investigate correlations among the N, P, and L proteins with NDV pathotypes (Table 2). The N_tail_ of NDV involves the interaction of the N-vRNA complex with the P protein and the binding of the N-vRNA template to the RNPs for vRNA synthesis [24, 37]. However, the specific aa sites or regions that determine these functions remain unclear.

**Table 2:** **Properties of recombinant NDVs harboring amino acid site, motif, or region mutation/exchange in the polymerase-associated proteins**

Viral protein	Virus	Parent	Amino acid site, motif, or region	Mutation/exchange^*^	MDT^†^	ICPI	Function	Reference
N	rSG10	SG10	E402	–	42 h	1.86	The N^E402^ residue negatively regulates the virulence of NDV by reducing the levels of vRNA synthesis early in virus infection	[71]
				E402A	36 h	1.81		
N	rI4 rHerts	I4 Herts/33	IDR(450–489)	– rI4[N_IDR(450–489)_]^Herts^	nd	1.96	The N_IDR(450–489)_ is essential for the oncolytic activity, replication ability, and virulence of NDV, and the N^L450^ rather than N^F450^ residue in IDR enhances the loading of viral mRNA onto ribosomes	[69]
					nd	1.90		
				– rHerts[N_IDR(450–489)_]^I4^	nd	1.65		
					nd	1.69		
L	rAV324	AV324/96	N1564V1694	–	nd	0.00	The L^N1564S^L^V1694E^ together with P^N37D^ mutations enhance the virulence, replication ability, and pathogenicity of pigeon-origin NDV due to the increased activity of viral RNA polymerase	[72]
				rAV324- L^N1564S^L^V1694E^	nd	0.18		
				rAV324- P^N37D^L^N1564S^L^V1694E^	nd	0.65		
L	rSG10	SG10	G-G-D motif K-D-K-E motif	–	48 h	1.81	The G-G-D and K-D-K-E motifs regulate the efficiency of viral mRNA translation and cell-to-cell spread ability of NDV, thus affecting viral virulence and pathogenicity	[78, 79]
				G1780A	52 h	1.66		
				G1782A	56 h	1.63		
				D1856A	48 h	1.60		
				K1756A	132 h	1.42		
				K1917A	108 h	1.48		
L	rG7	G7	K-D-K-E motif	–	44 h	1.60	The K-D-K-E motif is associated with the pathogenicity and immune evasion of NDV, and the L^E1954^ residue possibly inhibits the IFN-β production in host cells	[80]
				K1756A	120 h	0.14		
				D1881A	> 120 h	0.64		
				K1917A	> 120 h	0.30		
				E1954Q	> 120 h	0.30		

^*^indicates that no mutation/exchange is performed in the N and L proteins.

^†^nd, indicates that MDT is not detected.

A previous study indicated that the glutamic site at position 402 (E402) within the first intrinsically disordered region (IDR) of the N_tail_ negatively regulates NDV virulence by reducing vRNA synthesis. Recombinant NDV harbouring the N^E402A^ mutation displayed increased virulence and higher vRNA expression levels early in infection (Table 2) [71]. Furthermore, research involving exchanges of the second IDR (aa 450–489) of N_tail_ between oncolytic NDV [rHerts(N_IDR(450–489)_)^I4^] and non-oncolytic NDV [rI4(N_IDR(450–489)_)^Herts^] revealed that the N_IDR(450–489)_ region is crucial for oncolytic activity, replication efficiency, and cytopathogenicity. Notably, the N^L450^ residue, instead of the N^F450^ residue in this region, facilitates the loading of viral mRNAs onto cellular ribosomes (Table 2) [69].

Regarding the NDV P protein, limited studies have reported that the residues S48, T111, S125, and T271 are phosphorylated by protein kinase C. Particularly, the P/T111 residue plays a role in N-P interaction, as well as in viral replication and transcription [50]; however, its association with NDV pathotypes remains to be explored.

Research on a low-pathogenic NDV strain from pigeons that were passaged in chickens revealed that the mutations P^N37D^ and L^N1564S^L^V1694E^ contribute to enhanced virulence, replication, and pathogenicity due to increased activity of vRNA polymerase (Table 2) [72]. Other mutations of the residues in P^Q229H^ and L^F1676Y^L^F1844I^L^R2024H^ are also linked to increased pathogenicity of chicken-derived velogenic NDV in domestic ducks through serial passaging [73].

To date, several studies have shown that the MTase domain in the L protein, which contains the G-G-D and K-D-K-E motifs, is present in a wide range of paramyxoviruses, including NDV. This domain is essential in viral mRNA cap methylation and translation, affecting virus replication and pathogenicity [74–77]. Mutation experiments revealed that either the G-G-D motif or the K-D-K-E motif mutations significantly reduce the virulence and pathogenicity of recombinant NDVs by decreasing viral mRNA translation and the spreadability of NDV (Table 2) [78, 79]. Conversely, another study suggested that the attenuated virulence and pathogenicity observed in NDVs with the K-D-K-E mutation may result from reduced inhibition of IFN-β production in host cells (Table 2) [80]. This finding expands our understanding of the biological functions of the L protein’s MTase domain in the NDV life cycle.

In summary, the research highlights the identified aa sites, motifs, and regions in the polymerase-associated proteins that are associated with NDV pathotypes. These polymerase-associated proteins can act as multifunctional proteins and play various roles in the replication and pathogenicity of NDV.

## Correlation between polymerase-associated proteins and NDV thermostability

The most effective strategies currently used to prevent and control ND in poultry primarily rely on vaccination. However, there have been reports of vaccination failures with live lentogenic or attenuated ND vaccines due to issues related to storage and transportation [81–84]. As a result, thermostable live ND vaccine strains, such as V4, I2, TS09-C, and K148/08, have gained popularity and are widely utilized in many tropical developing and less-developed countries [85–88].

Traditionally used NDV live vaccine strains, like B1 and LaSota, are thermolabile, prompting increased interest in identifying the key factors contributing to these vaccines' thermostability. Recent advances using reverse genetics techniques have successfully elucidated the genetic basis related to NDV thermostability (Table 3). For instance, studies involving chimeric NDVs—created by exchanging viral genes between the thermolabile NDV LaSota and the thermostable NDV TS09-C—have revealed that the HN protein is a critical determinant of NDV thermostability. The chimeric NDV rLaSota(HN)^TS09^, which carries the thermostable TS09-C HN protein, exhibits a thermostable phenotype, and the reverse is also true (Table 3) [89].

**Table 3:** **The thermostability of recombinant NDVs harboring viral protein exchange**

Virus	Parent	MDT^*^	ICPI^†^	HA titer (log_2_)^‡^	Virus Titer (log_10_EID_50_/mL)	Thermostable phenotype	References
rTS09	TS09-C	> 168 h	0.00	9	9.50	Yes	[89]
rTS09(N + P + M)^LaSota^		nd	nd	nd	9.32	Yes	
rTS09(F + HN)^LaSota^		nd	nd	nd	8.95	No	
rTS09(F)^LaSota^		113 h	0.00	10	9.06	Yes	
rTS09(HN)^LaSota^		> 168 h	0.00	10	9.38	No	
rTS09(L)^LaSota^		nd	nd	nd	9.53	Yes	
rLaSota	LaSota	118 h	0.00	11	9.25	No	
rLaSota(N + P + M)^TS09^		nd	nd	nd	9.41	No	
rLaSota(F + HN)^TS09^		nd	nd	nd	9.04	Yes	
rLaSota(F)^TS09^		> 168 h	0.00	9	8.97	No	
rLaSota(HN)^TS09^		121 h	0.00	9	9.17	Yes	
rLaSota(L)^TS09^		nd	nd	nd	9.33	No	
rHR	HR09	62 h	nd	11	9.50	Yes	[90]
rHR(F)^LaSota^		104 h	nd	8	9.10	Yes	
rHR(HN)^LaSota^		73 h	nd	10	9.30	No	
rHR(N + P + M + L)^LaSota^		77 h	nd	9	9.30	Yes	
rLaSota	LaSota	112 h	nd	11	9.70	No	
rLaSota(F)^HR^		80 h	nd	9	8.90	No	
rLaSota(HN)^HR^		104 h	nd	10	9.50	Yes	
rLaSota(N + P + M + L)^HR^		109 h	nd	9	9.50	No	
rNDV4C	NDV4-C	> 120 h	0.00	9	8.625	Yes	[92]
rNDV4C(P)^Lasota^		> 120 h	0.00	9	8.625	No	
rLaSota	LaSota	> 120 h	0.00	10	9.375	No	
rLaSota(P)^NDV4C^		> 120 h	0.00	9	8.625	Yes	

^*,^^†,‡^nd, indicates that the MDT, ICPI, and HA titre are not detected, respectively.

The newly generated thermostable chimeric virus rLaSota(HN)^TS09^ retains its biological characteristics, inducing significantly higher antibody levels than TS09-C, providing complete protection against velogenic NDV infection [89]. A similar thermostable phenotype was observed in the chimera rLaSota(HN)^HR^, which contains the thermostable HR09 HN protein (Table 3). The residues P315 and V369 in the HR09 HN protein contribute to the thermostability of thermostable NDVs [90].

Moreover, research has shown that the HN and F proteins, particularly with the lentogenic NDV cleavage sites (^112^G-R/K-Q-G-R↓L^117^), influence NDV thermostability [91]. Further experiments demonstrated that the P protein also plays a role in thermotolerance; the chimera rLaSota(P)^NDV4C^, with the P protein from the thermostable NDV4-C, showed increased thermostability. Replacing the thermolabile LaSota P decreased the thermostability of chimera rNDV4C(P)^Lasota^ (Table 3). This finding underscores the importance of the P protein in determining NDV thermostability [92].

However, the relationship between the N and L proteins and NDV thermostability remains unclear. This ambiguity may stem from the significant biological or phylogenetic divergence observed in the gene-exchanged NDVs that will affect the genuine thermostability determinant differences. Furthermore, there have been too few virus gene replacement experiments to investigate NDV thermostability thoroughly. Overall, the current evidence indicates that the HN protein is the primary factor affecting NDV thermostability, with the F and P proteins also playing a notable role. Further studies are needed to clarify the relationship between polymerase-associated proteins and NDV thermostability.

## Interaction of NDV polymerase-associated proteins with host proteins

Research on virus-host interactions has long been a focus of study. This field not only uncovers the mysterious roles of viral proteins and the complex mechanisms behind viral pathogenicity [93–95] but also lays the groundwork for developing new antiviral drugs [96–98]. NDV, an essential representative of paramyxoviruses, has provided valuable insights into the unexplored replication and pathogenesis of paramyxoviruses [99–101].

**Figure 4:**
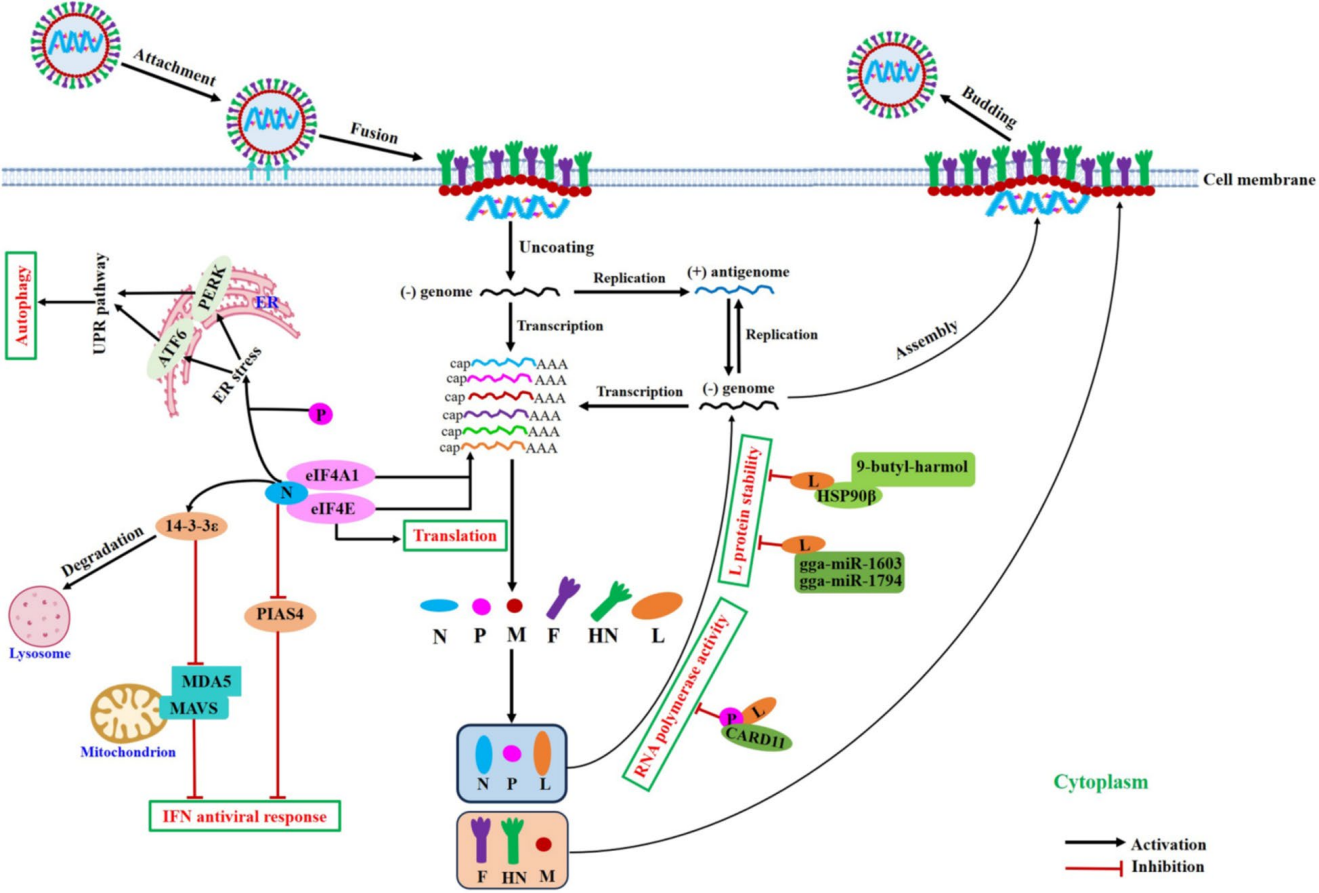
**Schematic representation illustrating the interaction between NDV polymerase-associated proteins and host proteins.** The NDV N protein interacts with the host eIF4E and eIF4A1 to promote the translation of viral mRNAs, and also interacts with 14-3-3ε and PIAS4 to enhance NDV replication by blocking the IFN antiviral response. Meanwhile, the NDV N or P protein activates the PERK and ATF6 pathways to induce autophagy and increase viral replication. In addition, the interaction between P protein and CARD11 inhibits the P-L interaction within the viral RNP complex, thus causing a reduction of RNA polymerase activity and inhibiting NDV replication. Moreover, host gga-miR-1603 and gga-miR-1794 specially target the NDV L gene to disrupt the stability of L protein and impede viral replication. Furthermore, 9-butyl-harmol disrupts the L-HSP90β interaction by directly targeting HSP90β, thereby decreasing the stability of NDV L protein and reducing NDV replication.

Many studies have reported on the interactions between NDV HN, M, and V proteins with host proteins [33, 62, 102–104]. However, there is comparatively little research on other NDV proteins. Given the crucial role of polymerase-associated proteins in NDV’s virulence, replication, and pathogenicity, recent studies have been increasingly focused on the interactions between the N, P, and L proteins and host proteins (Table 4 and Figure 4).

**Table 4:** **The functions of polymerase-associated protein-host protein interactions during NDV infection**

Viral protein	Interacting host protein	Interaction functions	References
N	eIF4A1	Leads to the inhibition of cellular mRNA translation with a complex 5' UTR structure and selectively promotes the translation of viral mRNAs	[69]
N	eIF4E	Facilitates the selective cap-dependent translation of viral mRNAs and enhances the synthesis of viral proteins by mutant K-D-K-E motif in the MTase domain of NDV L protein	[78, 99]
N	14–3-3ε	Mediates the degradation of 14–3-3ε to antagonise MAVS-mediated IFN antiviral response and promotes viral replication	[110]
N	PIAS4	Inhibits the expression of PIAS4 to block IFN antiviral response and enhances NDV replication	[111]
N or P	ATF6 PERK	Activates the PERK and ATF6 pathways to induce autophagy and increases the extent of NDV replication	[115]
P	CARD11	Inhibits the P-L interaction within the viral RNP complex, causing a reduction of vRNA polymerase activity and an inhibition of NDV replication; reduces syncytia formation and inhibits NDV replication through the CBM signalosome	[117, 119]
L	gga-miR-1603 gga-miR-1794	Facilitates the degradation of viral *L* genes at both the mRNA and protein levels to impede NDV replication	[120]
L	HSP90β	Increases the stability of the L protein to enhance NDV replication	[121]

A previous study demonstrated that NDV infection activates the p38 MAPK/Mnk1 and PI3K/Akt/mTOR pathways, promoting host cap-dependent translation. Notably, the interaction between NDV’s N protein and the eukaryotic translation initiation factor 4E (eIF4E) plays a significant role in the selective cap-dependent translation of viral mRNAs [99]. Additionally, the N-eIF4E interaction can be further enhanced by the mutant K-D-K-E motif within the MTase domain of the NDV L protein, which increases the replication, transcription, and translation levels of the NDV genome [78].

Another study found that the N protein of NDV, known for its strong oncogenicity, interacts with eukaryotic initiation factor 4A-I (eIF4A1) in the aa 366–489 region. This interaction inhibits cellular mRNAs' translation while selectively supporting viral mRNAs' translation [69]. These findings reveal new strategies by which NDV utilises the host’s translation machinery for viral protein synthesis.

Like most paramyxoviruses, NDV has developed various strategies to evade the host’s innate immune response. Current research highlights the immune evasion mechanisms associated with the viral V, W, and M proteins. However, whether NDV polymerase-associated proteins play a role in this process remains unclear.

14-3-3 family proteins contain seven isoforms that serve as functionally diverse signalling proteins [105, 106]. Notably, the 14-3-3ε isoform has been shown to play a crucial role in antiviral effects related to interferon (IFN)[107–109]. Recent studies have discovered that the NDV N protein interacts with the 14-3-3ε protein, prompting its entry into lysosomes for degradation. This interaction inhibits the binding of melanoma differentiation-associated protein 5 (MDA5) to mitochondrial antiviral signalling (MAVS), thereby blocking the IFN-mediated antiviral and facilitating increased NDV replication (Figure 4) [110].

Additionally, the protein inhibitor of activated STAT4 (PIAS4), which functions as a transcription factor that interacts with STAT and inhibits its activity, has been identified as another interacting factor of the NDV N protein [111]. Further analysis demonstrated that the NDV N protein decreases the expression of endogenous PIAS4. This reduction enhances viral replication by antagonising the antiviral IFN response [111].

Several studies have indicated that NDV can induce autophagy in cells and tissues, promoting viral replication [112–114]. Specifically, the NDV N or P proteins are sufficient to trigger endoplasmic reticulum (ER) stress, leading to autophagy through the activating transcription factor 6 (ATF6)- and PKR-like ER protein kinase (PERK)-related unfolded protein response (UPR) pathway (Figure 4) [115].

These findings shed light on the potential biological functions of the N and P proteins, thereby enhancing our understanding of NDV immune escape during viral pathogenesis.

The NDV L-P complex is crucial for vRNA synthesis and transcription [37]. Investigating host proteins that regulate the RNA polymerase activity of NDV is an intriguing direction for research. Caspase recruitment domain protein 11 (CARD11) is widely present in human and animal tissues [116]. The expression levels of chicken CARD11 (chCARD11) are significantly up-regulated in the brain tissue of chickens infected with avian neurotropic viruses, including NDV [117, 118].

Further studies have shown that chCARD11 directly interacts with the NDV P protein through its CC1 domain. The XD domain of the P protein, which mediates its interaction with the L protein, is also necessary for binding to chCARD11 [117]. Notably, the chCARD11 CC1 domain and the L protein compete to bind to the P protein via the XD domain. This competition inhibits P-L interactions within the viral RNP complex, reducing RNA polymerase activity and inhibiting NDV replication in neurons (Figure 4) [117].

Consistent with these findings, the P-chCARD11 interaction is also observed in a non-neural chicken embryo fibroblast cell line (DF-1 cells). In this context, chCARD11 can reduce syncytia formation and inhibit NDV replication through the CARD11-Bcl10-MALT1 (CBM) signalosome in DF-1 cells [119].

Recent advances have highlighted the importance of studying the L protein-host protein interactions, particularly their impact on protein stability and NDV replication. One study identified host microRNAs gga-miR-1603 and gga-miR-1794 as specific regulators of the NDV *L* gene. These microRNAs facilitate the degradation of the L gene at both the mRNA and protein levels, which in turn impedes NDV replication (Figure 4) [120].

Another study found that the NDV L protein, rather than the N and P proteins, interacts with heat shock protein 90β (HSP90β). This interaction increases the stability of the L protein, thereby enhancing NDV replication [121]. However, 9-butyl-harmol, an effective antiviral agent against paramyxovirus, disrupts the L-HSP90β interaction by directly targeting HSP90β. This disruption decreases the stability of the NDV L protein and reduces NDV replication (Figure 4) [121].

These findings deepen our understanding of NDV-host interactions and offer new avenues for antiviral therapies targeting polymerase-associated proteins to combat NDV infections.

## Future prospects

The *Paramyxoviridae* family is one of the most well-known and largest RNA virus families, posing significant threats to human and animal health. These viruses share a similar, though not identical, set of genes and protein functions. Currently, it is understood that the vRNA of all paramyxoviruses is encapsidated by the N protein, forming an NC that serves as the transcription and replication template for the L-P complex.

This similarity allows for beneficial comparisons between the structural and functional aspects of NDV and other paramyxovirus polymerase-related proteins. However, there are notable differences in the NC structure of the N protein, the tetrameric structure of the P protein, and the CD/MTase/CTD module of the L protein. Moreover, the mechanisms of initiation and elongation for the L-P complex in vRNA synthesis vary among NDV, PIV5, MuV, and HMPV [37, 40, 41, 52–54, 56]. These differences enrich the structural diversity of the paramyxovirus RNP complex and help clarify the functions of these various assemblies, which include RNA genome protection, transcription and replication, and encapsulation.

Despite significant progress in understanding the structural and functional properties of NDV polymerase-related proteins, research in this area remains less comprehensive than that of some other paramyxoviruses. Several questions still need to be addressed. For instance, the complete structure of the NDV P protein has not been determined, leaving the exact interaction between the P and N proteins unclear. Additionally, the vRNA genome is not included in the structure of L-P complex, and the entire complex containing L-P and N-vRNA-P has also not been observed due to the highly flexible nature of the P protein.

Fortunately, recent structural studies of the HMPV N-P interaction and the L-P-N-vRNA complex using cryo-EM in conjunction with molecular dynamics simulations have provided valuable insights [122–124]. These findings will serve as important references for further investigation into the structure and function of the RNP complex in NDV and other paramyxoviruses.

In recent years, studying the relationship between polymerase-associated proteins and viral virulence, pathotype, and thermostability has significantly enhanced our understanding of NDV replication and pathogenesis. While many studies have explored the functions of NDV polymerase-associated proteins, the role of complex protein–protein interactions between HN-M, M-N, N-P/V/W, and P-L in the replication and pathogenicity of NDV remains unclear.

Current evidence suggests that the W195 residue of the NDV V protein is crucial for its interaction with the N protein, which subsequently reduces vRNA synthesis, inclusion body formation, and overall viral replication and pathogenicity [125]. However, it is still not understood how other viral protein interactions may influence the RNA polymerase activity of NDV, or how these proteins intricately regulate the replication and transcription of the vRNA genome.

Based on existing research, only the NDV HN, F, and P proteins have been shown to play significant roles in thermostability. This indicates a need for further studies to clarify the exact functions of polymerase-associated proteins in NDV thermostability. Such insights could aid in developing more thermostable and efficient genotype-matched ND vaccines using reverse genetics techniques in the future.

Moreover, current research has primarily centred on interactions between host proteins and F, HN, M, and V proteins of NDV and other paramyxoviruses, with relatively little progress regarding host proteins that interact with polymerase-associated proteins. Recent studies have highlighted the importance of host protein interactions with NDV N, P, and L proteins. However, these investigations are still insufficient to fully elucidate the critical roles of polymerase-associated proteins in NDV replication and pathogenicity. This gap in knowledge limits the potential for developing novel antiviral drugs targeting these interactions for host-targeted therapies.

Consequently, exploring host proteins that restrict or limit NDV replication by targeting polymerase-associated proteins presents a promising avenue for research.

## Conclusion

In conclusion, we have systematically summarised and reviewed the structural features, virulence, pathotype, and thermostability correlation of NDV polymerase-associated proteins. We have also highlighted the essential roles of interactions between polymerase-associated proteins and host proteins in NDV replication and pathogenicity. While significant progress has been made in understanding both the unique and shared functions of NDV polymerase-associated proteins in recent years, research on the role of these viral proteins in replication and pathogenicity is still less comprehensive compared to that of other NDV proteins and most paramyxoviruses. Consequently, modern biotechnologies using gene modification, protein–protein interactome, multi-omics, and bioinformatic analysis studies, will provide new insights into the replication and pathogenesis of NDV. Overall, future efforts focused on the structural and functional studies of polymerase-associated proteins will undoubtedly enhance our understanding of the complex mechanisms underlying the replication and pathogenicity of NDV and related paramyxoviruses. This research also has the potential to contribute to the development of more effective vaccines and antiviral drugs against NDV and other paramyxovirus infections.

## Data Availability

Data sharing is not applicable to this article as no new data were created or analysed in this study.
